# Sex differences in metastatic surgery following diagnosis of synchronous metastatic colorectal cancer

**DOI:** 10.1002/ijc.34255

**Published:** 2022-08-31

**Authors:** Malin Ljunggren, Caroline E. Weibull, Gabriella Palmer, Emerik Osterlund, Bengt Glimelius, Anna Martling, Caroline Nordenvall

**Affiliations:** ^1^ Department of Molecular Medicine and Surgery Karolinska Institutet Stockholm Sweden; ^2^ Medical Unit of Trauma, Emergency Surgery and Orthopaedics Karolinska University Hospital Stockholm Sweden; ^3^ Clinical Epidemiology Division, Department of Medicine Karolinska Institutet Stockholm Sweden; ^4^ Department of Pelvic Cancer, GI Oncology and Colorectal Surgery Unit Karolinska University Hospital Stockholm Sweden; ^5^ Department of Immunology, Genetics and Pathology Uppsala University Uppsala Sweden

**Keywords:** colorectal neoplasms, neoplasm metastasis, sex, surgery, survival

## Abstract

The aim was to investigate gender differences in the likelihood to receive metastatic surgery, and to compare overall survival between men and women, among patients with synchronous metastatic colorectal cancer (mCRC) in a population‐based setting. All Swedish adult patients diagnosed with synchronous mCRC in 2007‐2016 were identified using the nationwide colorectal cancer database (CRCBaSe). Unadjusted and adjusted odds ratios (ORs) with 95% confidence intervals (CIs) were estimated using logistic regression, comparing the odds of receiving treatment. The Kaplan‐Meier method was used to calculate survival proportions and Cox regression models to estimate hazard ratios (HRs) and 95% CIs of all‐cause mortality rates. All multivariable models were adjusted for age, ASA score, Charlson comorbidity index, year of diagnosis, location of primary tumor and single or multiple metastatic locations. A total of 12 201 patients met the study criteria. Women received 23% less metastatic surgery for mCRC (adjusted OR = 0.77, CI:0.69‐0.86) and experienced a slightly higher mortality following diagnosis (adjusted HR = 1.09, CI:1.05‐1.14). In analyses restricted to patients who received metastatic surgery, no significant differences in mortality were found. In conclusion, this population‐based study showed that women less often received metastatic surgery of mCRC and experienced slightly higher all‐cause mortality compared with men. The differences persisted despite adjustments of patient and cancer characteristics. Gender differences in receiving treatment are unacceptable if the underlying explanation cannot be motivated. Further studies are needed to understand if the differences are based on sex (i.e., biology) or gender (including clinically unmotivated differences in treatment approach).

## Graphical abstract

What's new?

Gender differences in cancer therapy can have significant ramifications for patient outcome. Yet, why such differences exist and how extensively they impact patient survival are not fully known. Here, in Swedish patients with synchronic metastatic colorectal cancer (mCRC), the authors examined relationships between gender and odds of receiving metastatic surgery. Women were found to be less likely to have undergone metastatic surgery compared with men. Women also experienced higher all‐cause mortality. These differences were present even after adjustment for patient and cancer characteristics. The effect of gender on receiving metastatic surgery was modulated by hospital level and year of diagnosis.

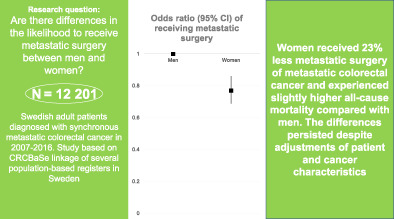

**Abbreviations**
ASA: American Society of Anaesthesiologists
CCI: Charlson comorbidity index
CI: confidence interval
cM: clinical metastasis
CRC: colorectal cancer
CRCBaSe: Colorectal Cancer Database Sweden
CRS‐HIPEC: cytoreductive surgery and hyperthermic intraperitoneal chemotherapy
HR: hazard ratio
ICD‐10: international classification of diseases 10
IPR: in‐patient register
mCRC: metastatic CRC
MDT: multidisciplinary team conference
OPR: out‐patient register
OR: odds ratio
OS: overall survival
pTNM: pathological tumor node metastasis
SCR: Swedish Cancer Register
SCRCR: Swedish Colorectal Cancer Register

## 1 INTRODUCTION

One in five patients diagnosed with colorectal cancer (CRC) have metastases at diagnosis.^1^, ^2^ Long‐term survival following a CRC diagnosis with liver, lung or peritoneal metastases is mainly dependent upon if treatment with palliative systemic therapy or curatively intended treatment including metastatic surgery can be given.^3^, ^4^, ^5^ Comparing all stages of CRC taken together, men have a higher incidence and mortality rate than women.^6^ Men also have a slightly higher recurrence rate and worse survival than women in nonmetastatic CRC.^7^, ^8^

The clinical presentation differs in men and women. Women develop CRC at a higher age,^9^ have less benefit from screening with flexible sigmoidoscopy.^10^ Right‐sided tumors are more common in women compared with men.^11^, ^12^, ^13^ Microsatellite instability (MSI)‐high and BRAF‐mutations^14^ are associated with poor prognosis in metastatic CRC (mCRC) and are more common in right‐sided tumors and thus also, in women. Right‐sided tumors are also associated with a higher age at diagnosis, more comorbidities, higher tumor grade and tumor stage and more peritoneal metastases.^13^ Thus, primary tumor location is an important factor to consider when studying differences in mCRC between men and women.

The potential association between gender and metastatic surgery or survival is not as clear. A national study from the Netherlands found that patients with left‐sided CRC and liver‐only metastases more often underwent metastasectomies than their right‐sided counterparts and had better survival. However, they did not find that gender influenced accessibility to treatment.^12^ Another study found that women were underrepresented in the group of patients that received surgical treatment for liver metastases but did not see an association between survival and gender when comparing the patients who received surgical treatment.^15^

Population‐based studies have shown gender differences in the treatment of several cancer diseases.^16^, ^17^, ^18^ Women receive less preoperative radiotherapy for rectal cancer, despite that men have higher postoperative mortality.^17^ We hypothesized that there were gender differences in the proportion undergoing metastatic surgery for mCRC that could not be explained by known confounding and mediating factors.

In this nationwide cohort of all Swedish patients with mCRC, the aim was to study gender differences in the likelihood to receive metastatic surgery (including locally ablative treatments) for synchronous liver, lung and peritoneal metastases of CRC origin. As a secondary aim, this study wanted to explore if there were any gender differences in overall survival (OS) following synchronous mCRC.

## 2 MATERIALS AND METHODS

The study was set in Sweden which has a decentralized tax‐funded universal healthcare system consisting of 21 regions. Primary colorectal cancer surgery is available in all regions while most surgeries for metastatic disease are centralized to Sweden's seven university hospitals. National guidelines on CRC care were introduced 2008 and initially stated that all patients should be investigated for metastatic disease and that patients with limited metastases should be considered potentially curable and discussed at a multidisciplinary team conference (MDT).^19^ In the 2016 update of the guidelines, it was recommended that all CRC patients were discussed at an MDT.^20^

### 2.1 The colorectal cancer data base (CRCBaSe)

The study was based on CRCBaSe (Colorectal Cancer Database Sweden), a recently established database where all patients identified in the Swedish Colorectal Cancer Register (SCRCR) have been linked to several other health and social population‐based registers, for example, the Swedish Cancer Register (SCR), the Cause of Death Register, and the In‐ and Out‐patient registers (IPR/OPR). CRCBaSe includes 76 955 individuals with a diagnosis of colon (registered since 2007) and/or rectal (registered since 1997) cancer registered in SCRCR until 2016. The coverage in SCRCR was 98.5% of colon adenocarcinomas and 98.8% of rectal adenocarcinomas during 2008‐2015.^21^ Because Swedish citizens and residents have unique personal identity numbers, linkage between different Swedish registers is possible.

### 2.2 Data extraction and definitions

To identify and classify the patients with synchronous metastases and retrieve data on treatment the SCRCR and IPR and OPR were used. The two latter are registers on all inpatient and outpatient specialist care visits in Sweden. Data on migration was retrieved from the Register of the Total Population. From the SCRCR, data was extracted on prevalence of synchronous lung and liver metastases, information on whether liver surgery was done at the same time as the primary tumor surgery, date of diagnosis, age, gender, American Society of Anaesthesiologists (ASA) score (mainly available if the patient's primary tumor was resected) and tumor data; that is, clinical metastasis (cM) stage, pathological tumor node metastasis (pTNM) stage, location of primary tumor, primary tumor resection and data on neoadjuvant therapy (unspecified if purpose was treatment of primary tumor and/or metastatic disease).

Information from the IPR and OPR was used to identify metastatic locations. The following international classification of diseases 10 (ICD‐10) codes were used to identify metastases; liver metastases: C787, lung metastases: C780, peritoneal metastases: C786, and other metastases: C770‐C779, C781‐C785 and C788‐C799. Data on metastatic surgery or locally ablative treatment of the metastases was also extracted from the IPR and OPR by use of surgical treatment codes, as described by the National Board of Health and Welfare in Sweden,^22^ that were registered 3 months before CRC‐diagnosis to 9 months thereafter on treatment of liver, lung and peritoneal metastases (see Table S1 regarding which codes were used to define metastatic surgery).

Charlson comorbidity index (CCI) was calculated for all patients based on data from the IPR, OPR and SCR.^23^ Data on previous CRC dating back to 1958 was retrieved from the SCR, to ensure that only patients with first‐time CRC were included. Data on deaths was retrieved from the Swedish Cause of Death register.

### 2.3 Study criteria

Patients aged 18 years or older with a primary diagnosis of CRC registered in SCRCR between 2007 and 2016 who had synchronous mCRC, either clinically assessed or pathologically confirmed, were included. Patients with a previous CRC registered in the SCR were excluded. Patients who did not have a specified metastatic location in SCRCR at the time of diagnosis or primary surgery, or a registered metastatic location in IPR/OPR 3 months before date of diagnosis to 9 months after were excluded.

### 2.4 Statistical methods

Patient characteristics were described using frequencies and proportions. Distributions between men and women were compared using the chi‐square test including missing values. The primary outcome was metastatic surgery (yes/ no). In addition, the proportion undergoing metastatic surgery restricted to patients discussed at preoperative MDT conferences was presented. The association between gender and metastatic surgery was evaluated using logistic regression models, yielding odds ratios (ORs) with 95% confidence intervals (CIs). Both unadjusted and adjusted models were fitted, where the latter were adjusted for age at diagnosis (continuous), ASA score, CCI, year of diagnosis (continuous), primary tumor location and number of metastatic locations (single, multiple). A potential interaction between gender and metastatic location was investigated and formally tested using the Wald test. Additionally, to evaluate potential gender differences by different types of metastases, interaction models were fitted estimating a separate OR for liver, lung, peritoneal metastases, other metastatic locations and combinations between any two or more of these.

The secondary outcome was OS. Patients were followed from date of CRC diagnosis until date of death, migration, or December 31, 2017, whichever occurred first. The Kaplan‐Meier method was used to estimate survival proportions for patients with mCRC, and in a separate analysis restricted to patients with liver metastases. The log‐rank test was used to test if the survival was significantly different. Cox regression models were used to estimate hazard ratios (HRs) with 95% CIs comparing the all‐cause mortality rate between males and females. Both univariable and multivariable models were fitted, where the latter were adjusted for age, ASA‐score, CCI, year of diagnosis, primary tumor location and number of metastatic locations. Missing data were handled using the missing‐indicator method. The proportional hazard assumption was formally tested using Schoenfeld residuals. Interaction models were fitted estimating separate HRs for different metastatic locations.

Sensitivity analyses were performed to investigate if the association between gender and metastatic surgery differed by certain subgroups of patients. Interaction models were fitted to yield separate ORs by age (18‐69/≥70 years), year of diagnosis (2007‐2011/2012‐2016), and hospital level (university/non‐university hospital). The age cut‐off was motivated by that biological age above 70‐75 years and high comorbidity are considered relative contraindications for some metastatic surgeries, for example, cytoreductive surgery and hyperthermic intraperitoneal chemotherapy (CRS‐HIPEC), according to Swedish national guidelines (16). For liver metastases, age above 70 years is considered a risk factor but not a contraindication. The interaction model with hospital level included patients diagnosed in 2009‐2016 since we lacked information on the registering hospital in 2007‐2008. All interactions were formally tested using the Wald test.

For the survival analyses, two additional Cox regression models were carried out. The first was restricted to patients who underwent metastatic surgery to evaluate potential gender differences in survival in this group. For this analysis the patients were followed from date of metastatic surgery (rather than diagnosis date). The second model was restricted to patients with liver metastases (liver only or in combination with other distant metastases), and compared the all‐cause mortality between men and women, with surgical intervention as an effect modifier. To avoid immortal time bias, this analysis was restricted to patients who survived 270 days after diagnosis (n = 5676), assuming they had then had the chance to be eligible for metastatic surgery. Follow‐up consequently started at the chosen landmark time point (diagnosis +270 days).

*P* values <.05 were considered statistically significant. All statistical analyses were done using Stata (StataCorp. 2017. Stata Statistical Software: Release 16. College Station, TX: StataCorp, LLC).

## 3 RESULTS

### 3.1 Patient characteristics

**FIGURE 1:**
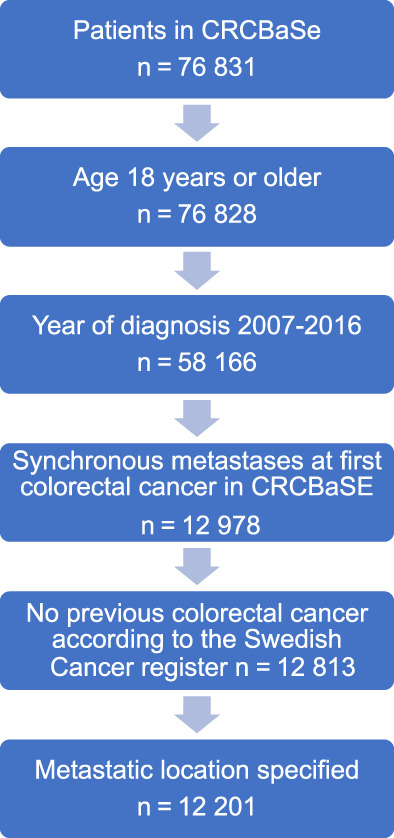
Flow chart showing selection of patient cohort according to study criteria. CRCBaSe, The colorectal cancer data base

**FIGURE 2:**
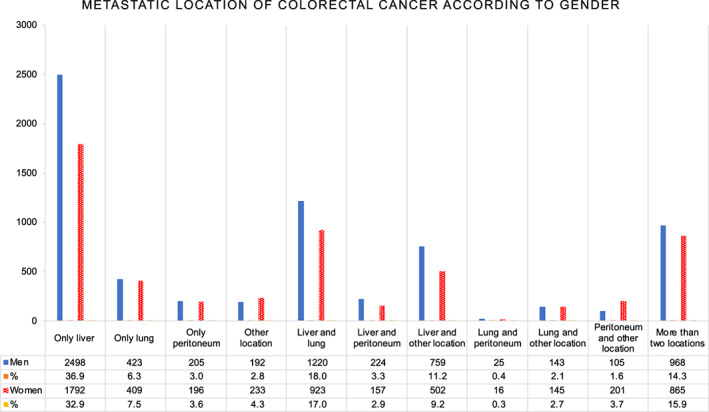
Metastatic location of 12 201 patients with synchronous metastatic colorectal cancer

There were 12 201 patients who met the study criteria (Figure 1). More men (n = 6762, 55%) than women (n = 5439, 45%) were diagnosed with synchronous mCRC. There was a similar proportion for whom the metastasis diagnosis was only clinical (cM1) in men (30%) and women (29%). The median age at diagnosis was similar between men and women, but a larger proportion of women were 80 years or older (Table 1). Right‐sided tumors were more common in women. There were statistically significant differences in metastatic sites (*P* value <.001). Men had liver‐only metastasis more often than women (n = 2498, 37% compared with n = 1792, 33%, *P* value <.001). Lung only and peritoneal only metastases were less common in men than in women (Figure 2). Women were less often discussed at pretherapeutic MDTs and received less neoadjuvant chemotherapy during the study period (Table 1).

**TABLE 1:** Patient and tumor characteristics of 6762 men and 5439 women diagnosed with synchronous metastatic colorectal cancer in Sweden 2007‐2016

		Men (n = 6762)	Women (n = 5439)	Chi‐squared *P* value
Age, median (range)	Years	70 (19–102)	71 (20–100)	
Age, categorized	18–64 years	2107 (31%)	1713 (31%)	*P* < .001
	65–79 years	3431 (51%)	2428 (45%)	
	≥80 years	1224 (18%)	1298 (24%)	
ASA score	1	429 (6%)	407 (7%)	*P* < .001
	2	1760 (26%)	1511 (28%)	
	3	1113 (16%)	935 (17%)	
	4–5	160 (2%)	93 (2%)	
	Missing^a^	3300 (49%)	2493 (46%)	
Charlson comorbidity index	0	4122 (61%)	3468 (64%)	*P* = .002
	1	713 (11%)	571 (10%)	
	2+	1927 (28%)	1400 (26%)	
Metastasis diagnosis	pM1 and cM1	4098 (61%)	3158 (58%)	*P* < .001
	pM1 only	635 (9%)	683 (13%)	
	cM1 only	2029 (30%)	1598 (29%)	
Year of diagnosis	2007–2011	3220 (48%)	2569 (47%)	*P* = .671
	2012–2016	3542 (52%)	2870 (53%)	
Primary tumor location	Right colon	1427 (21%)	1688 (31%)	*P* < .001
	Transverse colon	749 (11%)	754 (14%)	
	Left colon	2102 (31%)	1462 (27%)	
	Rectum	2439 (36%)	1492 (27%)	
	Unspecified colon	45 (1%)	43 (1%)	
Pretherapeutic MDT	No	1848 (27%)	1737 (32%)	*P* < .001
	Yes	4895 (72%)	3685 (68%)	
	Missing	19 (0%)	17 (0%)	
Neoadjuvant therapy, colon^b^	Chemotherapy	616 (14%)	477 (12%)	*P* = .005
	No	3599 (83%)	3388 (86%)	
	Missing	108 (2%)	82 (2%)	
Neoadjuvant therapy, rectum^b^	Chemotherapy	212 (9%)	110 (7%)	*P* = .106
	Radiotherapy	299 (12%)	196 (13%)	
	Radio‐chemotherapy	564 (23%)	308 (21%)	
	No	1294 (53%)	842 (56%)	
	Missing	70 (3%)	36 (2%)	
Primary tumor resection	No	3791 (56%)	2878 (53%)	*P* = .001
	Yes	2971 (44%)	2561 (47%)	
pT, resected tumors	1–3	1836 (62%)	1344 (52%)	*P* < .001
	4	1045(35%)	1137 (44%)	
	0/X	64 (2%)	60 (2%)	
	Missing	26 (1%)	20 (1%)	
pN, resected tumors	0	712 (24%)	546 (21%)	*P* = .028
	1	958 (32%)	797 (31%)	
	2	1200 (40%)	1119 (44%)	
	X	75 (3%)	81 (3%)	
	Missing	26 (1%)	18 (1%)	
Multiple metastatic locations	Single	3318 (49%)	2630 (48%)	*P* = .433
	Multiple	3444 (51%)	2809 (52%)	
Metastatic surgery	No	5504 (81%)	4579 (84%)	*P* < .001
	Yes	1258 (19%)	860 (16%)	
	➔Liver (only)	1042 (15%)	620 (11%)	
	➔Lungs (only)	75 (1%)	76 (1%)	
	➔Peritoneum (only)	111 (2%)	131 (2%)	
	➔Multiple locations	30 (0%)	33 (1%)	

Abbreviations: ASA, American association of anaesthesiologists; MDT, multidisciplinary team conference; pN, pathological node status; pT, pathological tumor status.

^a^ n = 5647 (97.5%) of patients with missing ASA data did not undergo primary tumor resection.

^b^ Unspecified if neoadjuvant therapy was for primary tumor and/or metastatic disease.

### 3.2 Metastatic surgery

Significantly more men (n = 1258, 19%) than women (n = 860, 16%) underwent metastatic surgery (*P* value <.001, Table 1). The difference remained after restricting the analyses to patients who had been discussed at an MDT conference at 18% for women compared with 21% for men (*P* value <.001). In all, women received 23% less metastatic surgery than men (OR = 0.77, 95% CI: 0.69‐0.86; Table 2). There was a significant interaction between gender and metastatic location (*P* from Wald test <.001), interpreted as the effect of gender on metastatic surgery was different for different metastatic locations. In the interaction model, women with solitary liver metastases received less metastatic surgery than men (OR = 0.75, 95% CI: 0.64‐0.89). For other metastatic locations there were no significant differences between men and women in the odds of metastatic surgery.

**TABLE 2:** Odds ratios (ORs) and 95% confidence intervals (CIs) comparing metastatic surgery rate between 12 201 men and women with synchronous colorectal metastases, estimated using logistic regression models

	Univariable model OR (95% CI)		Multivariable model^a^ OR (95 %CI)	
	Men	Women	Men	Women
Main effects model				
All sites	1.00	0.82 (0.75‐0.90)	1.00	0.77 (0.69‐0.86)
Interactions model by metastatic location				
Liver only	1.00	0.76 (0.66‐0.87)	1.00	0.75 (0.64‐0.89)
Lung only	1.00	0.97 (0.64‐1.47)	1.00	0.90 (0.58‐1.41)
Peritoneum only	1.00	0.69 (0.44‐1.09)	1.00	0.78 (0.46‐1.30)
Other metastases only	1.00	1.67 (0.41‐6.75)	1.00	1.63 (0.39‐6.75)
Multiple locations: Sensitivity analyses	1.00	0.98 (0.84‐1.13)	1.00	0.87 (0.74‐1.03)
Interaction model by age group^b^				
Age 18‐69 years	1.00	0.93 (0.83‐1.05)	1.00	0.84 (0.73‐0.97)
Age ≥ 70 years	1.00	0.70 (0.59‐0.82)	1.00	0.68 (0.57‐0.81)
Interaction model by diagnosis year				
2007‐2011	1.00	0.78 (0.67‐0.91)	1.00	0.73 (0.62‐0.86)
2012‐2016	1.00	0.85 (0.75‐0.96)	1.00	0.79 (0.69‐0.92)
Interaction model by hospital level^c^				
University hospital	1.00	0.95 (0.81‐1.12)	1.00	0.84 (0.69‐1.02)
Nonuniversity hospital	1.00	0.73 (0.63‐0.84)	1.00	0.70 (0.60‐0.82)

^a^ Adjusted for age (continuous), ASA, CCI, location of primary tumor, one or more metastatic locations and year of diagnosis (continuous except for in sensitivity analysis 2 where it was categorized; 2007‐2011, 2012‐2016).

^b^ The model included age categorized 18‐69/≥70 years as an effect modulator in addition to covariates listed above (*).

^c^ The model included 9968 patients diagnosed in 2009‐2016. Hospital level was included as an effect modulator in addition to covariates listed above (*).

### 3.3 Overall survival following diagnosis of mCRC

**FIGURE 3:**
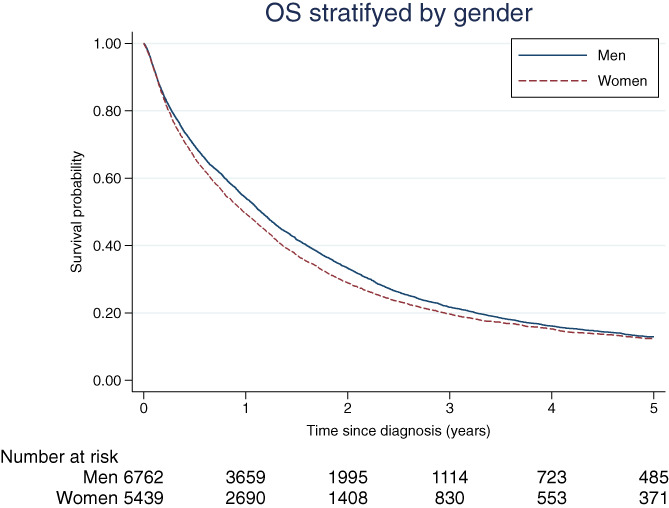
Kaplan‐Meier estimates of cumulative overall survival (OS) at 1, 3 and 5 years after diagnosis of stage IV colorectal cancer were lower for women compared with men: 49%, 20%, 12% and 54%, 22%, 13%, respectively (*P* value <.001)

Men with mCRC at diagnosis had superior OS to women (*P* value <.001). The 1‐year OS was 54% for men and 49% for women. The 5‐year OS was 13% and 12% for men and women, respectively (Figure 3). Men with liver metastases had superior OS to women (*P* value <.001) and the survival estimates followed a similar pattern as in the group with any metastatic location of CRC (Figure S1).

Women had a higher all‐cause mortality rate (adjusted HR = 1.09, 95% CI: 1.05‐1.14) after diagnosis of synchronous mCRC than men (Table 3). Women with solitary liver metastases had a 23% increased mortality rate compared with men (adjusted HR = 1.23, 95% CI: 1.15‐1.31). Moreover, women with liver metastases (solely or in combination with other metastatic locations) had a higher hazard of mortality compared with men (adjusted HR = 1.14, 95% CI: 1.09‐1.19). The effect of gender did not violate the proportionality assumption (*P* value from test of proportional hazards >0.05).

**TABLE 3:** Hazard ratios (HRs) and 95% confidence intervals (CIs) comparing all‐cause mortality between 12 201 men and women with synchronous colorectal metastases, estimated using Cox proportional hazards models

	Univariable model HR (95% CI)		Multivariable model^a^ HR (95% CI)	
	Men	Women	Men	Women
Main effects model				
All sites	1.00	1.09 (1.04‐1.13)	1.00	1.09 (1.05‐1.14)
Interactions model by one specified metastatic location or multiple locations				
Liver only	1.00	1.23 (1.15‐1.31)	1.00	1.23 (1.15‐1.31)
Lung only	1.00	1.00 (0.84‐1.18)	1.00	1.03 (0.87‐1.22)
Peritoneum only	1.00	1.05 (0.84‐1.31)	1.00	0.92 (0.73‐1.15)
Other metastases only	1.00	0.91 (0.73‐1.12)	1.00	0.94 (0.76‐1.16)
Multiple locations	1.00	1.03 (0.97‐1.08)	1.00	1.06 (1.01‐1.12)
Interactions model by liver metastases				
Liver metastases	1.00	1.14 (1.09‐1.19)	1.00	1.14 (1.09‐1.19)
No liver metastases	1.00	1.00 (0.91‐1.10)	1.00	1.01 (0.92‐1.10)
OS by metastatic surgery^b^				
No	1.00	—	1.00	—
Yes	1.00	1.02 (0.91‐1.16)	1.00	0.97 (0.85‐1.11)

^a^ Adjusted for age (continuous) ASA score, CCI, year of diagnosis (continuous), location of primary tumor and one or more metastatic locations.

^b^ A delayed entry approach was taken for patients undergoing metastatic surgery. There were 1956 patients that underwent metastatic surgery that are included in sensitivity analysis 4.

### 3.4 Sensitivity analyses

The effect of gender on odds of receiving metastatic surgery differed significantly by age, year of diagnosis and hospital level (all *P* value <.001). In both age groups, women received less metastatic surgery compared with men in the same age (adjusted OR = 0.84, 95% CI: 0.73‐0.97 for ages 18‐69 years, and adjusted OR = 0.68, 95% CI 0.57‐0.81 for ages ≥70 years; Table 2). The odds of undergoing metastatic surgery were slightly more decreased for women compared with men during 2007‐2011 (OR = 0.73, 95% CI: 0.62‐0.86), than during 2012‐2016 (adjusted OR = 0.79, 95% CI: 0.69‐0.92). Women first managed by nonuniversity hospitals had 30% decreased odds of undergoing metastatic surgery compared with men (adjusted OR = 0.70, 95% CI: 0.60‐0.82).

The Cox regression model restricted to patients with liver metastases who survived 270 days after diagnosis (Table S2) showed that there was a statistically significant interaction between gender and metastatic surgery (*P* from Wald test <.001). Among patients who did not have metastatic surgery (n = 3966), there was a statistically significant difference in all‐cause mortality between women and men (adjusted HR [95% CI]: 1.08 [1.01‐1.16]). There were no significant gender differences in mortality rates for patients who received metastatic surgery (Table 3).

## 4 DISCUSSION

This nationwide study of 12 201 patients with synchronous mCRC showed that men and women are treated differently. Women received 23% less metastatic surgery, a difference that persisted after adjustment for differences in patient and tumor characteristics. Women were discussed at pretherapeutic MDTs less often and received neoadjuvant treatment less often for colon tumors, indicating that the whole chain‐of‐treatment from diagnosis to metastatic surgery is affected by patient gender. Moreover, factors not associated with biology, for example, year of diagnosis and managing hospital modulated the effect of gender on receiving metastatic surgery, which indicates that the observed differences cannot solely be explained by biological differences. Furthermore, women experienced a higher mortality rate. Interestingly, this gender difference was not seen among patients who received potentially curative treatment of the metastases.

### 4.1 The findings in relation to previous research

The association between gender and treatment is in accordance with previous publications on both upper and lower gastrointestinal cancers.^16^, ^17^, ^18^ Women undergoing elective surgery for oesophageal adenocarcinoma receive less neoadjuvant treatment than men.^18^ Women receive less preoperative radiotherapy for rectal cancer even despite the fact that men have higher postoperative mortality.^17^ Women with gastric and oesophageal adenocarcinomas receive less palliative systemic treatment and have poorer OS.^16^

To our knowledge this is the first nationwide study that found gender differences in an unselected group of patients with synchronous mCRC and the chance of metastatic surgery. Liver metastases were more common in men, as has been shown previously.^24^ However, when comparing patients with liver metastases only, women received 25% less liver metastasectomies. These numbers are comparable with previous published numbers on liver surgery from Great Britain and Sweden that showed that women were 23% and 26% less likely to undergo liver resection, respectively.^25^, ^26^ A Dutch study found no association between gender and receiving CRS‐HIPEC, nor of gender and survival.^27^ In line with these results, there were no gender differences in treatment of peritoneal metastases in this study. Women had their primary tumors resected to a slightly higher degree than men. A reason for this could be that women had more right‐sided tumors, which are associated with a higher risk of bowel obstruction in patients aged 65 years or older,^28^ and of emergency resection.^29^

### 4.2 Differences in tumor biology as possible mediators

Women have a higher proportion of right‐sided tumors which are associated with worse prognosis if the disease is metastatic.^12^, ^30^, ^31^ Peritoneal metastases,^12^, ^32^, ^33^ *BRAF* V600E mutations,^33^ and MSI‐high tumors^33^, ^34^ are more common in patients with right‐sided tumors compared with left‐sided tumors and they are all factors associated with worse prognosis in mCRC.^33^, ^34^, ^35^ The presence of *BRAF* mutations,^36^ and probably also MSI‐high tumors, are associated with poorer survival after liver resection, and these factors are considered prior to the decision to recommend liver surgery or not. Since the register does not have information about molecular properties, it is not possible to investigate potential gender differences in molecular characteristics independent of primary location.

This study did not have information on the extent of metastatic tumor burden. However, to our knowledge, no study has described any gender differences in tumor burden in patients with mCRC. In a recent study on curatively treated patients with CRC with peritoneal metastases there were no gender differences in tumor burden (information from the author).^37^ Another study showed that primary tumor location affected the burden of liver metastases,^38^ a factor adjusted for in all multivariable models. In the present study, there was no gender difference in the presence of multiple metastatic locations, which is in line with a previous study.^39^ To decrease the mediating effect of differences in tumor location and its associated tumor biology and metastatic pattern, all analyses were adjusted for tumor location. The associations between gender and metastatic surgery, and mortality still remained.

### 4.3 Differences in attitudes and civil status as potential mediators

Differences in patients' and clinicians' behaviors and attitudes are other possible mediating factors. This study has information on whether the patient received metastatic surgery or not, but not on whether they were recommended and declined metastatic surgery. Previous studies have not shown gender differences in doctor's delay in referral^40^ or in prolonged patient‐attributable delay of primary CRC diagnosis.^40^, ^41^, ^42^ There are studies that show that socioeconomic factors, for example, education and living alone are associated with the chance of receiving metastatic surgery and combination chemotherapy for mCRC.^43^, ^44^ It is possible that gender differences in socioeconomic status may partly explain the observed differences in given treatment. Nonetheless, gender differences that can be explained by socioeconomic differences are not acceptable in a healthcare system striving to provide equal care based on patients' needs.

### 4.4 Future studies

The underlying explanation for the observed gender differences in metastatic surgery is probably multifactorial. The logistic regression interactions models showed that factors not associated with biology, for example, year of diagnosis and managing hospital modulated the effect of gender on receiving metastatic surgery, which indicates that the observed differences cannot solely be explained by biological differences. Further studies, with detailed clinical and molecular information, are needed to understand if a proportion of the gender differences in treatment is clinically motivated. If the gender differences are not fully clinically motivated, additional research is needed to understand which parts of given care that are haltering. This should include efforts to understand if it is the clinicians that recommend metastatic surgery to fewer women than men, or if it is the female patients that decline treatment recommendations to a higher extent than men.

### 4.5 Strengths and weaknesses

The main strength of this study is its nationwide design including a large, unselected material of patients who are diagnosed with synchronous mCRC, making the results generalizable to countries with a similar population and health‐care system. Furthermore, we had detailed information on comorbidities and metastatic location, making it possible to adjust for confounders and effect modifiers. The patients were prospectively registered in the SCRCR, a register with a very high coverage and detailed data on patient, tumor and surgical treatment characteristics.^8^, ^21^ Because the CRCBaSe includes information from the patient registers as well as SCRCR, the presence and treatment of metastatic disease could be verified, generating a high internal validity.

The main weakness of the study is its limitation to register data, and as in all observational studies there is a risk of residual confounding. The reasons why the patient did or did not undergo metastatic surgery were unknown. In addition, this study lacked information on tumor load and mutational status and cannot explore whether this might contribute to gender differences in given treatment. Another limitation is the lack of information on chemotherapy type and frequency, and targeted therapies and response to these, which are other important factors that affect survival following mCRC. Female sex is associated with more chemotherapy toxicity but this does not affect efficacy in terms of OS or disease‐free survival in mCRC.^45^, ^46^ More extensive information on oncological treatment and treatment response could have taken us further in our analysis regarding the possible causes to the gender differences observed in this study.

## 5 CONCLUSIONS

This population‐based study revealed that women with mCRC less often underwent metastatic surgery, predominantly when liver metastases were present, and had a slightly impaired survival compared with men. The differences persisted after adjusting for known confounders and differed by year of diagnosis and hospital level. Gender differences in receiving treatment are unacceptable if the underlying explanation cannot be motivated. Further studies are needed to investigate whether these gender differences are biologically motivated or due to clinically unmotivated differences in treatment approach.

## AUTHOR CONTRIBUTIONS

The work reported in the paper has been performed by the authors, unless clearly specified in the text. Author contributions according to the CRediT taxonomy: Malin Ljunggren: conceptualization, methodology, formal analysis, writing—original draft. Caroline E. Weibull: conceptualization, methodology, software, data curation, writing—review & editing. Gabriella Palmer: conceptualization, writing—review & editing. Emerik Osterlund: conceptualization, writing—review & editing. Bengt Glimelius: conceptualization, writing—review & editing. Anna Martling: conceptualization, writing—review & editing, resources, funding acquisition. Caroline Nordenvall: conceptualization, methodology, resources, writing—review & editing, supervision, funding acquisition.

## FUNDING INFORMATION

The project was supported financially by the Swedish Cancer Society, the Stockholm Cancer Society, the Regional agreement on medical training and clinical research between the Stockholm County Council and Karolinska Institutet (ALF‐project). None of the funders have had any role in the current study.

## CONFLICT OF INTEREST

Dr. Weibull is a member of the Board of Directors of Red Door Analytics and has a current employment at War On Cancer. She is part of a research collaboration between Karolinska Institutet and Janssen Pharmaceutica NV for which Karolinska Institutet has received grant support. This was unrelated to this study. The other authors declare no competing interests.

## ETHICS STATEMENT

The study was approved by the Regional Board of the Ethical Committee in Stockholm, Sweden (DNR: 2014/71‐31, 2018/328‐32, 2021‐00342). The need to obtain informed consent was waived.

## Supporting information


**TABLE S1** Treatment codes as described by the National Board of Health and Welfare in Sweden used to identify curative intended treatment of metastases, translated from Swedish.
**TABLE S2** Hazard ratios (HRs) and 95% confidence intervals (CIs) comparing all‐cause mortality between 5676 men and women with synchronous colorectal liver metastases by surgical intervention, estimated using Cox proportional hazards model
**FIGURE S1** Kaplan‐Meier estimates of cumulative overall survival (OS) from diagnosis of liver metastatic synchronous colorectal cancer

## Data Availability

Questions regarding the data availability should be addressed to the board of CRCBaSe (email: crcbase@mmk.ki.se). Further information is available from the corresponding author upon request.
