# Supplementary material for: Sex differences in metastatic surgery following diagnosis of synchronous metastatic colorectal cancer
Source: Int J Cancer. 2022 Aug 31;152(3):363–73. doi: 10.1002/ijc.34255 (PMC10086966; doi:10.1002/ijc.34255)

Supplementary material

Title: Sex differences in metastatic surgery following diagnosis of synchronous metastatic colorectal cancer

Authors: Malin Ljunggren, Caroline E Weibull, Gabriella Palmer, Emerik Osterlund, Bengt Glimelius, Anna Martling, Caroline Nordenvall

Table of contents:

Page 1: Title page

Page 2: Supplementary table 1

Page 3: Supplementary table 2

Page 4: Supplementary figure 1

Supplementary Table 1. Treatment codes as described by the National Board of Health and Welfare in Sweden used to identify curative intended treatment of metastases, translated from Swedish.

**Liver**	
JJA40	Excision of growth in the liver
JJA41	Laparoscopic excision of growth in the liver
JJA43	Destruction of growth in the liver
JJA44	Laparoscopic destruction of growth in the liver
JJA96	Other local procedure of the liver
JJA97	Other laparoscopic local procedure of the liver
JJB-	Liver resections
JJW96	Other procedure of liver
TJJ10	Percutaneous destruction of growth in liver
DJ026	Percutaneous transluminal injection of Yttrium-90 microspheres in the liver
**Lungs**	
GDA20	Extirpation of local growth in the lungs
GDA21	Thoracoscopic extirpation of local growth in the lungs
GDB-	Lung resections
GDC-	Lung lobectomies
GDD-	Pulmectomies
ZV520	Stereotactic radiation (non-intracranial)
**Peritoneum**	
JAL20	Extirpation or destruction of peritoneal growth
JAW96	Other surgery on abdominal wall, mesentery, peritoneum or omentum.
JAQ00	Extensive extirpation of peritoneum, total or subtotal extirpation at certain metastatic conditions.
JAQ10	Intraoperative hyperthermic chemotherapeutic washing of the abdominal cavity. Adjuvant therapy after extensive extirpation of the peritoneum.

Supplementary Table 2. Hazard ratios (HRs) and 95% confidence intervals (CIs) comparing all-cause mortality between 5,676 men and women with synchronous colorectal liver metastases by surgical intervention, estimated using Cox proportional hazards model

	Univariable model HR (95% CI)		Multivariable model* HR (95% CI)	
	Men	Women	Men	Women
	Univariable model	Multivariable model*	Univariable model	Multivariable model*
Interaction model by metastatic surgery restricted to patients with liver metastases				
No metastatic surgery	1.00	1.08 (1.01-1.16)	1.00	1.08 (1.01-1.16)
Metastatic surgery	1.00	1.03 (0.68-1.18)	1.00	1.02 (0.89-1.18)

*Adjusted for age (continuous) ASA score, CCI, year of diagnosis (continuous), location of primary tumour and one or more metastatic locations.

Supplementary Figure 1. Kaplan-Meier estimates of cumulative overall survival (OS) from diagnosis of liver metastatic synchronous colorectal cancer.

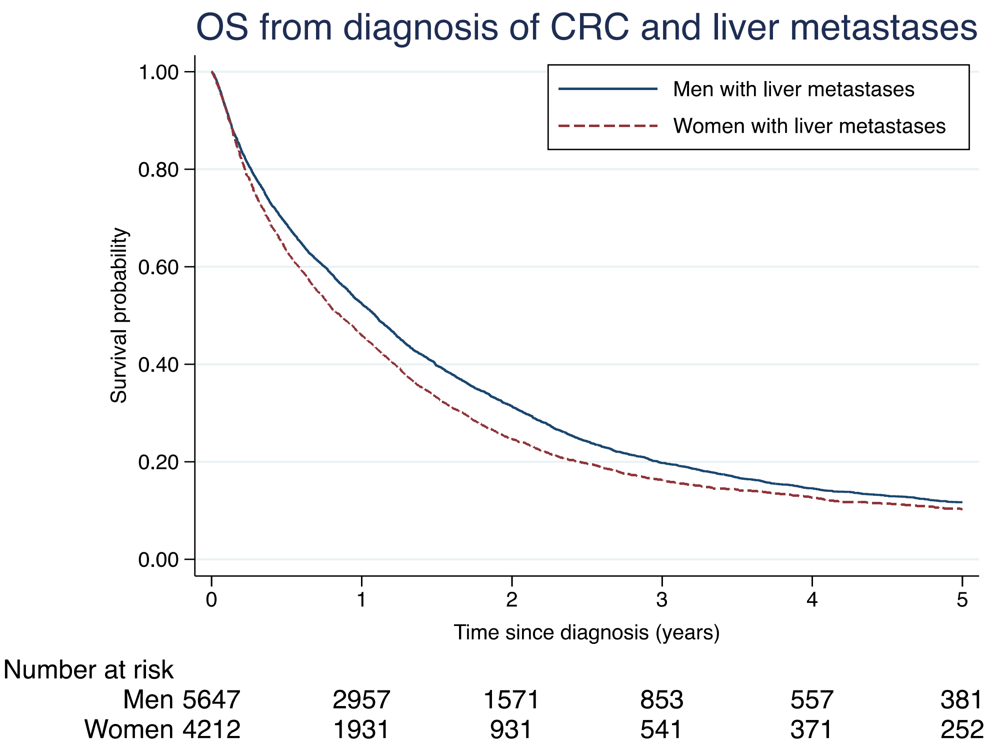

Supplement: Supplementary file 1 — TABLE S1 Treatment codes as described by the National Board of Health and Welfare in Sweden used to identify curative intended treatment of metastases, translated from Swedish. TABLE S2 Hazard ratios (HRs) and 95% confidence intervals (CIs) comparing all‐cause mortality between 5676 men and women with synchronous colorectal liver metastases by surgical intervention, estimated using Cox proportional hazards model FIGURE S1 Kaplan‐Meier estimates of cumulative overall survival (OS) from diagnosis of liver metastatic synchronous colorectal cancer [file IJC-152-363-s001.pdf]
